# Correction to “*N*-Nitrosodimethylamine Formation in Metformin Drug Products by the Reaction of Dimethylamine and Atmospheric NO_2_”

**DOI:** 10.1021/acs.oprd.3c00473

**Published:** 2024-01-03

**Authors:** Shohei Fukuda, Kanako Kondo, Shoji Fukumoto, Norimichi Takenaka, Osamu Uchikawa, Itsuro Yoshida

In Figure 1 of the published article, the structure of metformin hydrochloride was incorrect. The corrected figure is given here.

**Figure 1:**
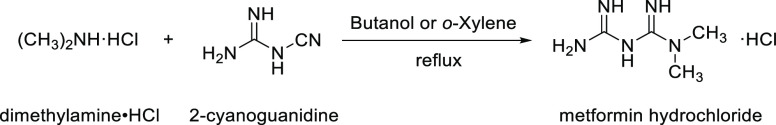
Preparation of metformin hydrochloride.

